# Synthesis and Characterization of Activated Carbon Co-Mixed Electrospun Titanium Oxide Nanofibers as Flow Electrode in Capacitive Deionization

**DOI:** 10.3390/ma14226891

**Published:** 2021-11-15

**Authors:** Gbenro Folaranmi, Myriam Tauk, Mikhael Bechelany, Philippe Sistat, Marc Cretin, Francois Zaviska

**Affiliations:** Institut Européen des Membranes, IEM, UMR-5635, Université de Montpellier, ENSCM, CNRS, Place Eugène Bataillon, CEDEX 5, 34095 Montpellier, France; gbenro.folaranmi@umontpellier.fr (G.F.); myriam.tauk@umontpellier.fr (M.T.); mikhael.bechelany@umontpellier.fr (M.B.); philippe.sistat@umontpellier.fr (P.S.)

**Keywords:** flow electrode capacitive deionization, electrospinning, activated carbon, desalination

## Abstract

Flow capacitive deionization is a water desalination technique that uses liquid carbon-based electrodes to recover fresh water from brackish or seawater. This is a potential second-generation water desalination process, however it is limited by parameters such as feed electrode conductivity, interfacial resistance, viscosity, and so on. In this study, titanium oxide nanofibers (TiO_2_NF) were manufactured using an electrospinning process and then blended with commercial activated carbon (AC) to create a well distributed flow electrode in this study. Field emission scanning electron microscope (FESEM), X-ray diffraction (XRD), Raman spectroscopy, X-ray photoelectron spectroscopy (XPS), and energy dispersive X-ray (EDX) were used to characterize the morphology, crystal structure, and chemical moieties of the as-synthesized composites. Notably, the flow electrode containing 1 wt.% TiO_2_NF (ACTiO_2_NF 1 wt.%) had the highest capacitance and the best salt removal rate (0.033 mg/min·cm^2^) of all the composites. The improvement in cell performance at this ratio indicates that the nanofibers are uniformly distributed over the electrode’s surface, preventing electrode passivation, and nanofiber agglomeration, which could impede ion flow to the electrode’s pores. This research suggests that the physical mixture could be used as a flow electrode in capacitive deionization.

## 1. Introduction

One of the growing challenges of the 21st century is the availability of fresh water. Water contamination because of anthropogenic activities such as industrialization, demographic change, and global warming has resulted in a significant increase in demand for safe drinking water. Water desalination technology could help to alleviate this problem by delivering high quality, pure water. Most desalination technologies, such as multiple effect desalination (MED), reverse osmosis (RO), and others, have high capital costs (when considering plant setup) and energy consumption (when considering pre- and post-treatment of water, as in RO), necessitating the development of a new desalination technique [1].

Capacitive deionization (CDI) is a growing desalination technology attracting attention as an energy-efficient, cost-effective, and ecofriendly water treatment technology. The first and most widely used CDI form involves a pair of porous carbon electrodes separated by a space in which salt water flows as an influent perpendicular to the applied electric field direction [2]. A fundamental variation of this basic CDI form emerged with the unfolding of membrane CDI (MCDI). In this architecture, ion exchange membranes were added to the CDI cell configuration to block co-ions from carrying parasitic current, which improves charge efficiency and can increase the charge storage in the electrodes porous structure [3]. In the past decade, a new class for CDI based on MCDI was developed which introduced carbon flow electrodes that can be pumped through the electrode compartments. Flow electrode CDI (FCDI) is a promising second-generation water desalination method based on the principle of ions adsorption. When a specific voltage is applied, it entails using a polarized flow electrode (liquid electrode) travelling via a flow channel to adsorb ions from brackish or seawater via electrical double layer (EDL) formation (interface between electrolyte and electrode) [4].

One of the challenges in FCDI is to improve the conductivity and capacitance of carbon materials used as electrodes, as well as reducing the resistance between the electrode and the electrolyte. This can be accomplished by improving the surface charge of activated carbon for better EDL formation [5].

Because of its beneficial qualities such as high surface area, porosity, availability, and low cost, graphitic carbon, materials such as activated carbon are frequently used as flow electrodes [6,7]. However, for more effective EDL formation and better ion storage, the resistance at the electrode-electrolyte interface should be lowered. As a result, surface modification is required to raise the surface charge of activated carbon.

Metal oxides such as zinc oxide (ZnO), tin oxide (SnO), zirconium oxide (ZrO), and titanium oxide (TiO_2_) have been used to modify carbon in this concept [8,9]. Among all these oxides, TiO_2_ has a high surface charge that can be used in conjunction with AC to create new types of electrodes. The aggregation of TiO_2_ particles, however, is one of the additive’s downsides, limiting its performance [10,11,12]. Small quantities of TiO_2_ nanofibers are used in activated carbon flow electrodes to alleviate the agglomeration problem since they have a favorable large axial ratio morphology [13,14].

Due to the high resistance of the liquid medium utilized for carbon dispersion for slurry formation, charge transfer resistance is a strong factor hindering the ease of ions diffusion into the pores of carbon electrodes in flow electrodes [15]. As a result, methods for reducing interfacial resistance by adding conducting additives or introducing oxygenated functional groups to activated carbon have been reported in the literature [15,16,17,18], but no report on the properties of TiO_2_ nanofibers in reducing interfacial resistance and their effect on the rheological properties of carbon-based flow electrodes has been found to date.

Nanofiberous materials of high surface area and porosity are considered recently to be used for generating sustainable and green energy resources. Electrospinning method is a low-cost method used to create nanofiberous materials suited for many energy-related applications such as supercapacitors, and Li-ion batteries [19,20].

Motivated by this, we have synthesized TiO_2_ nanofibers by electrospinning process and mixed it with commercial AC. The proposed electrodes which consisted of different weight percentage of TiO_2_NF were labelled TiO_2_NF-x where x represents the weight percentage of TiO_2_NF in the composite (x = 0.5, 1.0, 1.5, 2.0, 2.5, and 5.0 wt.% TiO_2_NF). The electrodes were characterized by FESEM, XRD, Raman spectroscopy, XPS, EDX, and N_2_ adsorption/desorption. Dynamic viscosity of the mixtures in suspension to build the flow electrodes was also investigated. Hence, the electrochemical properties of the as-prepared flow electrodes (AC and TiO_2_NF-x) were analyzed by cyclic voltammetry (CV) to determine their capacitance after which the flow electrodes were tested in a laboratory-made FCDI cell for desalination.

## 2. Materials and Methods

### 2.1. Materials

Activated carbon, Darco (CAS no: 7440-44-0, MW 12.01 g mol^−1^), titanium (IV) isopropoxide (C_12_H_28_O_4_Ti, CAS no: 546-68-9, MW 284.22 g mol^−1^, 97%), poly(vinyl pyrrolidone) (PVP, CAS no: 9003-39-8, MW 1,300,000 g mol^−1^), and sodium chloride (NaCl, CAS no: 7647-14-5, MW 58.44 g mol^−1^, 99%) were obtained from Sigma Aldrich, Steinheim, Germany. Acetic acid (CH3COOH MW 60.052 g mol^−1^, 99.5% assay), absolute ethanol (C2H5OH MW 46.07 g mol^−1^, 99.99%,) were obtained from VWR chemicals, Fontenay-sous Bois, France. Polyvinylidenefluoride (PVDF) (CAS no: 24937-79-9) was obtained from Alfa Aesar, Erlenbachweg 2, Kendel, Germany. All reagents were used without any further purification. Cationic and anionic exchange membranes were purchased from Membranes International Inc. (Ringwood, NJ, USA) and Deionized water (18 MΩ·cm) was used to prepare standard solutions and suspensions.

#### 2.1.1. Synthesis of TiO_2_ Nanofibers by Electrospinning

The TiO_2_ nanofibers were synthesized by electrospinning technique. Briefly, solution A containing 3 mL of ethanol was added to 0.3 g of polyvinyl pyrrolidone (PVP) and then stirred until dissolution. Then, solution B consisting of 2 mL ethanol, 2 mL of acetic acid, and 3 mL of titanium isopropoxide was stirred for 45 min. This is then followed by the addition of solution B into clear solution of A and then further stirred for 50 min to obtain a sol gel solution. Electrospinning of the sol–gel solution was carried out at 22.30 kV. The distance between the collector and the syringe tip was maintained at 10 cm, and the injection speed was 0.5 mL min^−1^. The obtained electrospun nanofiber materials were sintered in air atmosphere at 400 °C for 4 h with a ramp rate of 1 °C min^−1^.

#### 2.1.2. Preparation of AC and ACTiO_2_NF–x Flow Electrodes

For flow electrode preparation, certain amount of powdered commercial AC and ACTiO_2_NF-x (in which x corresponds to 1.0, 1.5, 2.0, 2.5, and 5.0 wt.% of TiO_2_NF) were weighed and dispersed in deionized water as presented in Table 1. The mixtures were sonicated for 3 h and stirred for 1 h before being fed into the cell. The tank containing the slurry electrode was continuously stirred on a magnetic stirrer during the course of the experiment.

### 2.2. Physical Characterization

FESEM was used to analyze the morphology of the samples (Hitachi S4800, Tokyo, Japan). The structural properties were studied by using Raman spectroscopy (HORIBA Xplora, Tokyo, Japan) and XRD (Pan Analytical X’pert Phillips, Almelo, The Netherlands). XPS (ESCALAB 250 Thermo Electron, Montigny Le Bretonneux, France) and EDX (X-Max, Oxford, UK) were used to investigate the atomic composition and chemical moieties of the materials. For the XPS analysis, the excitation source was a monochromatic source Al Kα anode with photoenergy that was observed at 1486.6 eV. The analyzed surface has a diameter of 500 µm. The photoelectron spectra were calibrated in terms of bond energy with respect to the energy of the C=C component of carbon C1s at 284.4 eV. Surface area was obtained by using N_2_ adsorption/desorption at 77 K (Micromeritics ASAP, Verneuil en Halatte, France). Dynamic viscosity was measured using Anton Paar Rheometer Physica MCR 301 (Anton Paar, Graz, Austria).

### 2.3. Electrochemical Characterizations

Solid electrodes for electrochemical characterization of pristine AC and ACTiO_2_NF were made by combining activated carbon powder (0.32 g), carbon black (0.04 g), and poly (vinylidene fluoride fluoride PVdF, 0.04 g) in 3 mL N-Methyl-2-pyrrolidone (NMP). To establish homogeneity, the mixture was agitated for 2 h and then sonicated for 40 min. After that, the slurry was applied on a graphite sheet. The coated electrode was dried in an oven for 1–2 h at 80 °C. The as-synthesized titanium oxide nanofibers were added (0.5, 1.0, 1.5, 2.0, 2.5, and 5.0 wt.%) to AC containing carbon black and PVDF, and the mixture was agitated for several hours in 3 mL NMP for ACTiO_2_NF synthesis. After that, the mixture was sonicated for 40 min. The slurry was subsequently immobilized by depositing it on a graphite sheet. It was then dried in an oven at 80 °C for 1–2 h to produce solid electrodes.

The electrochemical properties of the prepared electrodes were examined by using CV. CV tests were performed using a three-electrode system. The mixtures were deposited on a graphite sheet as support with an exposed surface area of 1 cm^2^, while a platinum mesh and a saturated 3 M KCl, Ag/AgCl electrode served as counter and reference electrodes respectively. A molar NaCl solution was used as electrolyte. Voltammetry measurements were performed with Origalys potentiostat (OGF01A, Origalys Electrochem SAS, Rillieux-la-Pape, France) at an operating window from −0.4 to 0.6 V vs ref (to ensure electrochemical stability of the electrolyte and prevent water splitting i.e., oxygen and hydrogen evolution) in 1 M NaCl electrolyte.

The double-layer capacitance was determined using cyclic voltammetry at different scan rates by considering the charging and the discharging currents at 0.1 V vs. ref. The determined double-layer capacitance of the system was the average of the absolute value of the slope of the linear plot of charging and discharging currents fitted to the data. CDL as the double layer capacitance was determined using Equation (1):

i = υ C_DL_. (1)

The double-layer charging current density i (A·cm^−2^) is equal to the product of the scan rate υ (V·s^−1^), and the electrochemical double-layer capacitance C_DL_ (F·cm^−2^).

### 2.4. FCDI Measurement

**Figure 1:**
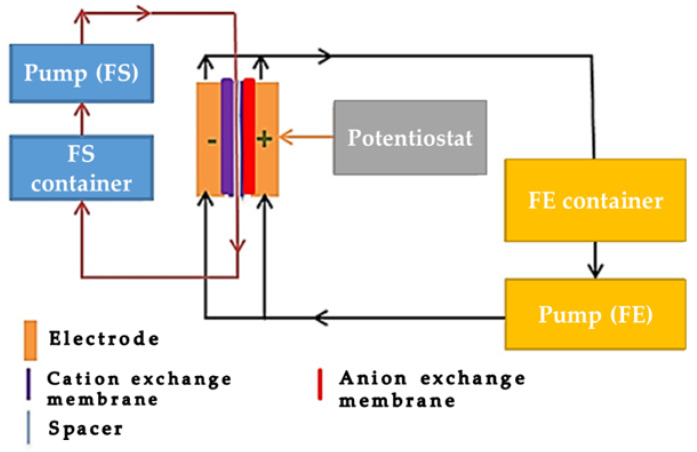
Schematic diagram of FCDI setup is a figure.

**Figure 2:**
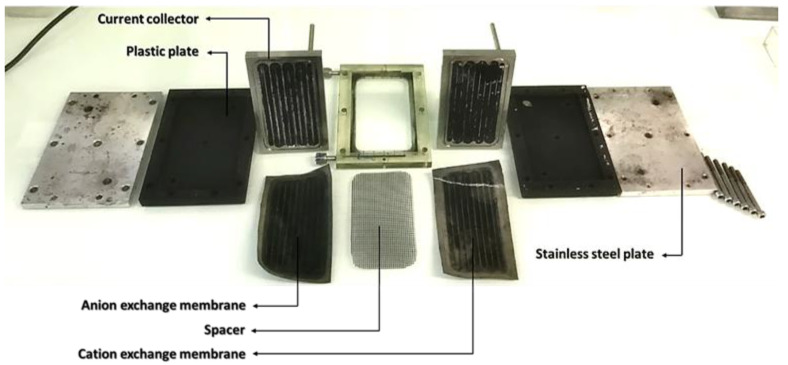
Individual components of FCDI cell.

The schematic diagram of the close loop experimental set-up is shown in Figure 1 in which the cell was powered by a potentiostat and the feed solution (FS, 5 g·L^−1^) was made to pass through a spacer sandwiched in between cation and anion exchange membranes. The feed electrodes (FE) stored in a reservoir (were made to pass (by pumping) through flow channels and as they exit the channels, they are fed back to the reservoir and then re-circulated; this allows co-mixing of opposite charged ions outside the cell. Figure 2 shows the breakdown of the cell components with the current collector (6 mm width and 0.9 mm depth channel), the ions exchanges membranes and spacer (0.9 mm thick). The flow rate of the electrodes was operated at 40 mL min^−1^. Each desalination experiment was conducted for 30 min. The initial conductivity of the salt solution and that of the effluent was monitored at room temperature by ion conductivity meter (Hanna Instruments SRL) all along the 30 min of the experiment. A constant voltage difference of 1.2 V was applied to the FCDI unit cell using an Origalys potentiostat (OGF01A, Origalys Electrochem SAS) for desalination experiments. The current intensity passing through the FCDI unit cell was consequently measured by the potentiostat during the experiment.

In the present work, the following indicators defined FCDI performances:

Salt removal rate in mg·min^−1^·cm^−2^ (SRR) relates to the mass of salt adsorbed (mg) per FE-FS contact area (cm^2^) per unit of time (min). It is calculated by Equation (2):

(2) $$\mathrm{SRR}=\frac{\left(\mathrm{Co}-\mathrm{Cf}\right)*V}{A*t}$$

Co and Cf are the initial and final (at t = 30 min) concentration (mol·L^−1^), respectively, V is the volume of the solution (L), A is the contact area between FE: FS, and t is the charging time.

The salt removal efficiency in % (SRE) was calculated using Equation (3):

(3) $$\mathrm{SRE}=\frac{\left(\mathrm{Co}-\mathrm{Cf}\right)}{\mathrm{Co}}*100$$

Charge efficiency (CE) in % which relates to the ratio of salt adsorbed to the quantity of charge passed into the system was calculated by Equation (4):

(4) $$\mathrm{CE}=\frac{z\;\left(\mathrm{Co}-\mathrm{Cf}\right)\;V\;F}{M\;\int Idt}*100$$

where z is the equivalent charge of the ions, F is the Faradaic constant, M is the molar weight, and ∫I*d*t is the integrated quantity of charge passed to the system as a function of time. CE is a good indicator for the energy efficiency of the system and will directly affect the operating cost of the system (OPEX).

## 3. Results and Discussion

### 3.1. Morphology

**Figure 3:**
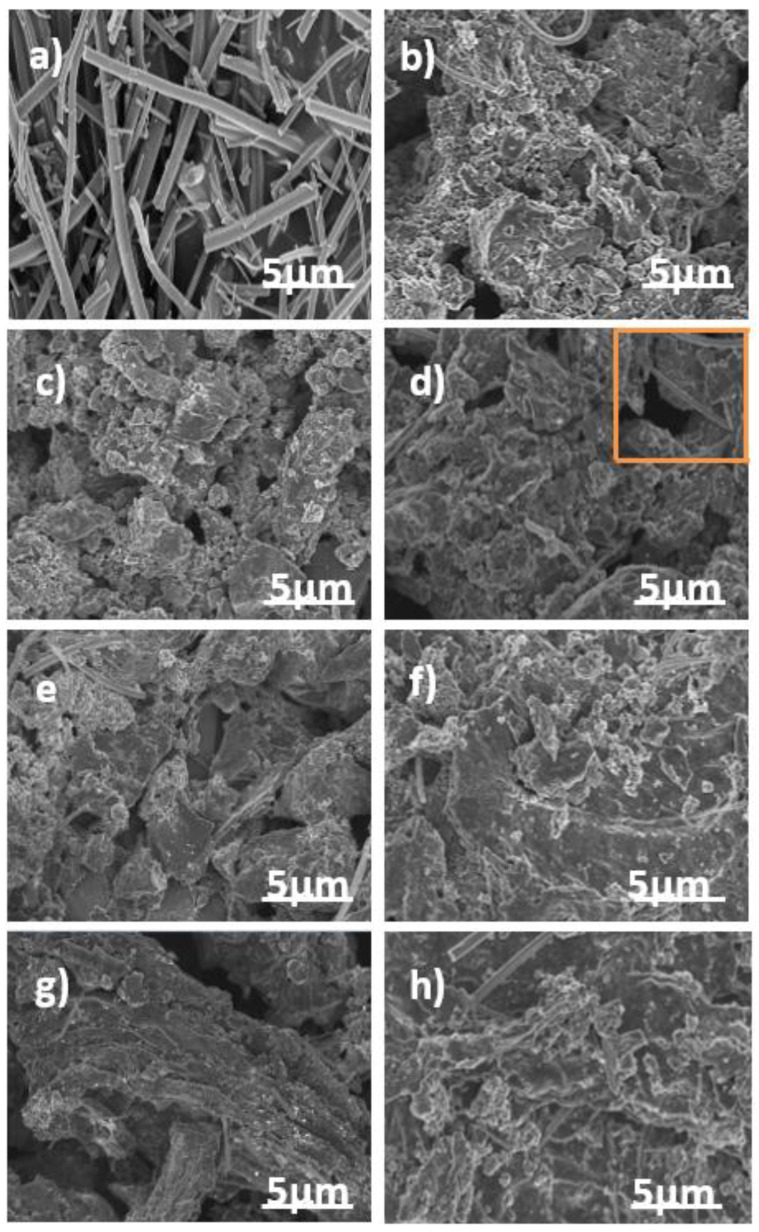
FESEM top view images of (**a**) TiO_2_NF (**b**) AC (**c**–**h**) ACTiO_2_NF–x, where (x = 0.5, 1.0, 1.5, 2.0, 2.5 and 5.0 wt.% TiO_2_NF) respectively.

The morphology of as-synthesized titanium oxide nanofibers (TiO_2_NF), pristine AC, and the ACTiO_2_NF-x electrodes are shown in Figure 3a–h respectively. From Figure 3a, it is apparent that the electrospun TiO_2_ showed fiber-like morphology with no beads formation. This shows the successful nanofibrous morphology of TiO_2_ formation by electrospinning process. Figure 3b shows the morphology of pristine AC. It is clear that it has no defined shape with rough or uneven surface characteristics while Figure 3c–h reveals the presence of TiO_2_NF on the surface of AC, indicating that the additive was successfully introduced by co-mixing.

### 3.2. Structural Properties

**Figure 4:**
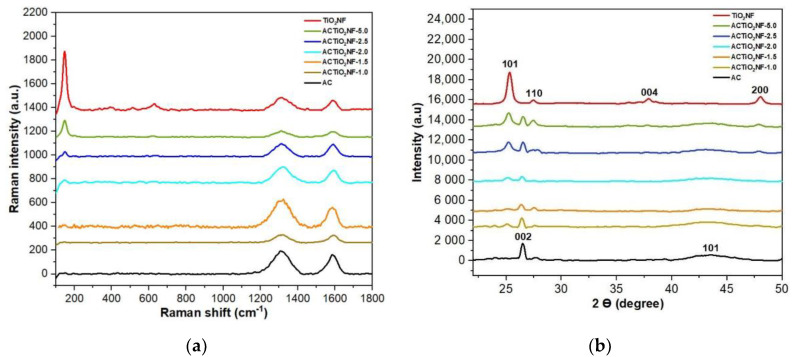
(**a**) Raman spectra of Titanium oxide nanofibers TiO_2_NF, pristine AC, and its composites (ACTiO_2_NF–x) and (**b**) diffractograms of titanium oxide nanofibers (TiO_2_NF), pristine AC and its composites (ACTiO_2_NF–x where x = 0.5, 1.0, 1.5, 2.0, 2.5, and 5.0 wt.% TiO_2_NF).

Figure 4a,b shows the Raman spectra and diffractogram of the TiO_2_NF, pristine AC and ACTiO_2_NF s respectively. The Raman spectrum of the pristine AC and ACTiO_2_NF in Figure 4a conforms to a typical graphitic carbon with distinguishable peaks at 1350 cm^−1^ and 1590–1610 cm^−1^ corresponding to D and G bands respectively [21]. D band arises from a defect that is based on out of plane vibration while G band relates to the ordered structure of graphite crystals [13]. For the as-synthesized TiO_2_NF, Figure 4a, major peaks are observed at 142, 388, 516, and 638 cm^−1^ indicating anatase phase characteristics [8]. Distinguishable peaks of TiO_2_NF were observed in some of the composites especially for those with high percentage of TiO_2_NF as shown in Figure 4a. This indicates the successful mixing of the nanofibers with the AC.

Structural investigation was carried out to understand the crystalline nature of the materials. Figure 4b shows the diffractogram of pure TiO_2_NF, pristine AC, and its composites. The TiO_2_NF crystals are predominantly dominated with definite and sharp diffraction peaks at 2θ = 25°, 39° and 43.5° relating to 101, 004, and 200 planes of anatase phase respectively with the presence of rutile phase at 2θ = 27.5°, 36°, and 41° relating to 110, 101, and 111 planes respectively [8]. Typical diffraction peaks of all graphite material is observed for pristine AC and ACTiO_2_NF at 2θ = 26° and 43.5 ° corresponding to 002 and 100 or 101 planes of graphite respectively. The sharp diffraction peaks observed at 002 planes indicates the presence of graphite microcrystalline structure in the AC [13]. In comparison with pristine AC, peaks of TiO_2_NF were detected at 25°, 27.5°, and 48° diffraction peaks of TiO_2_NF in all the composites thus showing successful doping of the nanofibers in the AC. Furthermore, using Debye-Scherrer equation, (D = Kλ/β, cosθ) where K is the constant value of 0.9, λ is the radiation of the XRD machine (0.1541 nm), β is the full width at half maximum of the diffraction peak in radian, and θ is the diffraction angle in radian, no changes were observed in the crystallite size (10 ± 0.830 nm) of the as-synthesized TiO_2_ nanofibers and ACTiO_2_NF.

### 3.3. EDX and XPS Studies

**Figure 5:**
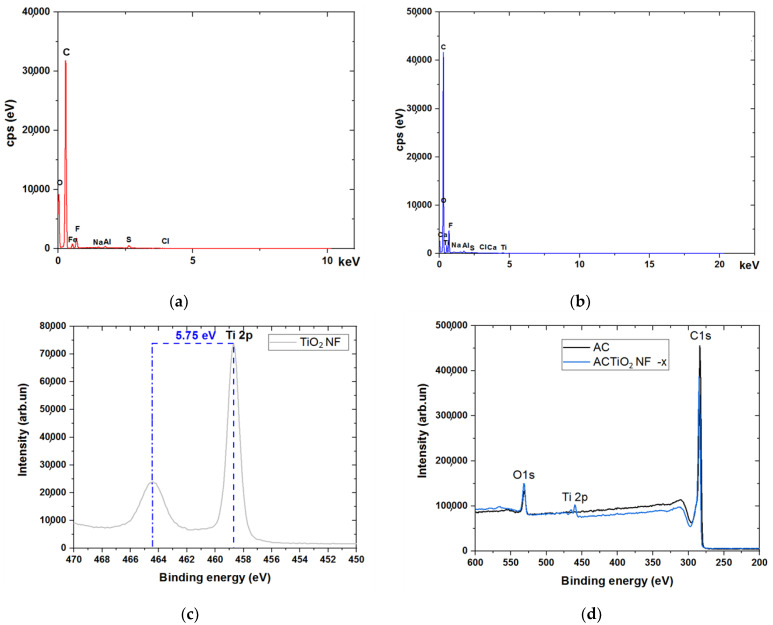
EDX spectra of (**a**) pristine AC (**b**) ACTiO_2_NF–x and XPS spectra of (**c**) Ti 2p for TiO_2_NF (**d**) the composite pristine AC and ACTiO_2_NF where x = 1.0 wt.% TiO_2_NF.

Figure 5a,b shows the EDX spectra that were obtained in order to identify the composition of the pristine AC and the composite electrode. Evidently from EDX, the elements detected at highest percentage in our materials are C and O. Titanium was detected among other elements in little quantity in the composite as shown in Figure 5b. The presence of fluorine was also detected due to the addition of PVDF (binder) added during electrode fabrication. Moreover, EDX mapping was further used to investigate the distribution of the additive in the mixture. From Figure S1a,b it can be seen that the additive distribution at lower concentration (1 wt.%) is homogenous (even dispersion) while at higher concentration (5 wt.%), its distribution is concentrated within a particular region. The results confirm the formation of well-dispersed nanofibrous within the carbon structure at low percentage concentration.

Further investigation was carried out using XPS in order to verify any change in the chemical composition of the pristine AC and its composites. Figure 5c shows two prominent peaks at 458.69 and 464.44 eV belonging to Ti 2P_3/2_ and Ti 2P_1/2_ respectively [22,23]. From Figure 5c, it is shown that Ti (IV) is present in normal state in the composites due to the observed spin orbital splitting corresponding to 5.76 eV that is obtained between Ti 2P_1/2_ and Ti 2P_3/2_. [23]. As shown in Figure 5d, no titanium was detected in the XPS spectra survey scan of the pristine AC on comparison with ACTiO_2_NF; a complementary result with that of EDX. The O1s peak in the composite increased a little bit when compared to pristine AC due to the influence of oxygen content of the additive. According to XPS, the atomic composition of the ACTiO_2_NF-1.0 consisted of C1s 90.6 ± 0.11%, O1s 7.9 ± 0.12%, and Ti 2p 1.0 ± 33.33% while that of pristine AC is C1s 94 ± 0.84% and O1s 5 ± 3.84%.

### 3.4. Rheology Study

**Figure 6:**
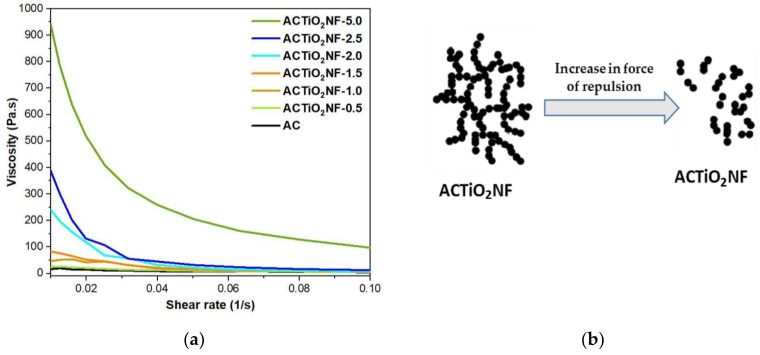
(**a**) Rheological properties of the flow-electrodes. (**b**) Schematic diagram of the ACTiO_2_NF mixture behavior under repulsive forces.

Rheology property reveals the flow nature of the slurry used under applied force. The viscosity nature of the slurry electrode was measured as a function of shear rate. Here, rheology property is in terms of dynamic viscosity, which is used to describe resistance to flow of liquid while shear rate describes the speed of deformation of the slurry under applied force. The dynamic viscosity was determined at a constant concentration of 10 wt.% carbon content in the slurry. From Figure 6a, it is obvious that the slurry follows a non-Newtonian fluid (shear thinning effect) in which the viscosity of the slurries decreases with increasing shear rate. To understand the effect of the additive, the viscosities of both pristine AC and the ACTiO_2_NF mixture were measured and compared as shown in Figure 6a. It is obvious from the rheogram curves that viscosity increases as the additive content increases in the composite. However, at low percentage of additive, there seems to be no significant difference in the viscosities of both the pristine AC and the ACTiO_2_NF mixture but at i.e., TiO_2_NF ≥ 2.0 wt.%, a sharp increase in viscosity was observed. The increase in viscosity can be attributed to the high specific surface area (increase frictions) of titanium nanofibers as well as its surface charge. The surface charge of the nanofibers (whether positive or negative) leads to creation of repulsive forces existing within the nanoparticles and as a result, they tend to move further apart due to strengthened force of repulsion; consequently, viscosity increases with increasing repulsive forces [24,25,26,27,28]. Therefore, as the concentration of the additive in the electrodes increases, a distinguishable jump in their viscosity was observed due to increase in force of repulsion as shown in Figure 6b. This implies that the presence of additive at high content level could lead to potential clogging of the feed electrodes in the cell.

### 3.5. Electrochemical Properties

Electrochemical behavior of the pristine AC electrode and ACTiO_2_NF electrodes were carried out using CV at different scan rates in a potential window from −0.4 to 0.6 V vs. Ref (to ensure electrochemical stability of the electrolyte and prevent water splitting i.e., oxygen and hydrogen evolution). The experiment was conducted in 1 M NaCl aqueous solution to investigate the influence of the additive at different ratios. CV is an important technique to probe the capacitive nature of EDL [29,30].

**Figure 7:**
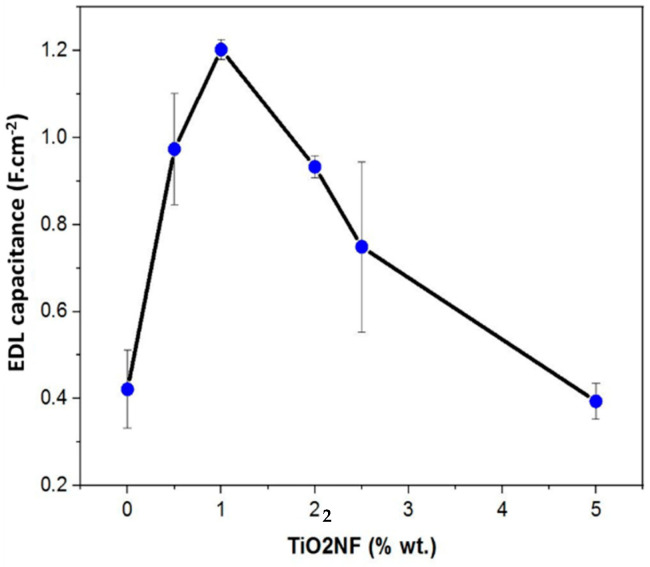
Electrical double-layer capacitance of the system for AC and ACTiO_2_NF-x composite electrodes (ACTiO_2_NF–x where x = 0.5, 1.0, 2.0, 2.5, and 5.0 wt.% TiO_2_NF).

Based on the cyclic voltammetry cycles, the EDL capacitance of the electrodes was calculated [31] and is shown in Figure 7.

Thus, incorporating TiO_2_NF at low rate into our carbon material enhances its double layer capacitance due to the formation of a uniform network distribution of TiO_2_ nanofibers between AC particles. However, as the presence of TiO_2_NF increases in the composite, there seem to be exhibition of poor electrochemical behavior possibly due to the increase in resistance to easy flow of ions into the pores of the electrode because of TiO_2_NF agglomeration [14]. Our results correlate to the findings reported in literature [8,14].

### 3.6. Desalination Performance

**Figure 8:**
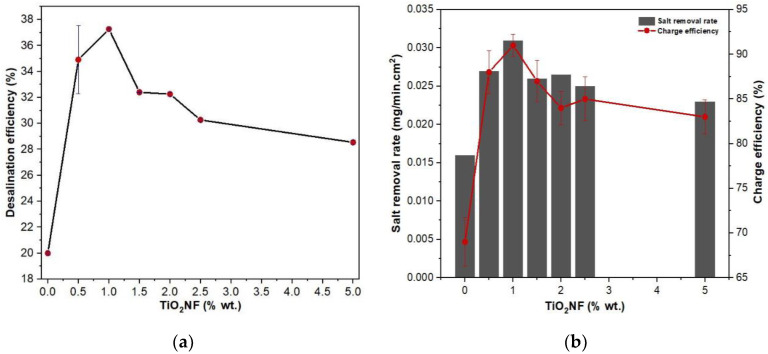
(**a**) Desalination efficiency; (**b**) salt removal rate and charge efficiency of AC and ACTiO_2_NF−x, where (x = 0.5, 1.0, 1.5, 2.0, 2.5, and 5.0 wt.% TiO_2_NF) respectively.

As reported in literature, the electrosorption performance of carbon-based materials is linked to their capacitive properties amid other factors [9,29]. As explained earlier, TiO_2_NF was added at different wt.% to influence the capacitance of commercial AC and this factor was verified through the electrochemical characteristics of the electrodes. Furthermore, in order to confirm the link between electrosorption performances and capacitive properties, desalination experiments were carried out under a semi-continuous system in a flow channel. Desalination was conducted at an operational cell potential ΔE = 1.2 V for 30 min using 5000 mg·L^−1^ NaCl as the feed solution. Difference in conductivity was monitored and recorded during the experiment. FCDI performance indicators such as desalination efficiency (DE) in Figure 8a, salt removal rate (SRR), and charge efficiency (CE) in Figure 8b were used to evaluate the performance of the flow electrodes.

Important increase in DE is noticed in Figure 8a. Notably, the ACTiO_2_NF electrodes show higher DE than pristine AC and among all the electrodes, ACTiO_2_NF-1.0 exhibited the best DE, which implies fast ion mobility to the pores of the flow electrode thus leading to quick salt removal; a consequent effect on CE and SRR.

Low desalination behavior of ACTiO_2_NF-x (x ≥ 2.0 wt.% TiO_2_NF) at high percentage could be due to the fact that at this ratio, the nanofibers tend to agglomerate among themselves (not well dispersed or less uniform) and as such, making the pores of the electrode (surface hindrance) not easily accessible for ions adsorption. In addition, electrode passivation is likely to occur due to the high presence of the nanofibers [32]. This will make the electrode surface not easily permeable for ions (impermeable layer formation on electrode surface) thus significantly affecting the electrosorption performance of the electrodes as observed in our materials, (whitish layers on the surface of flow electrodes). It has to be mentioned that only a small fraction of titanium nanofiber (≤1% weight) is sufficient to significantly increase FCDI performances without increasing much viscosity.

## 4. Conclusions

To summarize, we used an electrospinning procedure to create titanium nanofibers. Without any further post-treatment, the as-spun nanofibers were rationally mixed with AC using a simple agitating procedure to generate hybrid composites of ACTiO_2_NF-x (x = 0.5, 1.0, 1.5, 2.0, 2.5, and 5.0 wt.% ACTiO_2_NF). The composites were subsequently described and tested for the first time as electrodes in FCDI. Introduction of TiO_2_ nanofibers into AC improved the electrochemical characteristics of the material. The addition of TiO_2_ nanofibers to the composite electrodes increased their performance, with ACTiO_2_NF-1.0 demonstrating distinguishing and remarkable properties with the best desalination behavior. The composites’ nanofibrous shape allows for greater anchoring within the AC network, allowing for improved ion transport and migration to the pores. Finally, the technology described has the potential to produce carbon-based flow electrodes with better shape and desalination performance in the FCDI methodology.

## Figures and Tables

**Table 1:** Composition of flow electrodes.

Electrode Material	FE (10 wt.%) (g)	DH_2_O (mL)	TiO_2_NF (g)
AC	7.80	70	0
ACTiO_2_NF-0.5	7.76	70	0.04
ACTiO_2_NF-1.0	7.72	70	0.08
ACTiO_2_NF-1.5	7.68	70	0.12
ACTiO_2_NF-2.0	7.64	70	0.16
ACTiO_2_NF-2.5	7.60	70	0.20
ACTiO_2_NF-5.0	7.40	70	0.40

Note: FE: Feed electrode; DH_2_O: deionized water.

## Data Availability

The data presented in this study are available on request from the corresponding authors.

## Supplementary Materials

The following are available online at https://www.mdpi.com/article/10.3390/ma14226891/s1. Figure S1: EDX mapping of (a) ACTiO_2_NF-5.0 and (b) ACTiO_2_NF-1.0.
