# Supplementary material for: Synthesis and Characterization of Activated Carbon Co-Mixed Electrospun Titanium Oxide Nanofibers as Flow Electrode in Capacitive Deionization
Source: Materials (Basel). 2021 Nov 15;14(22):6891. doi: 10.3390/ma14226891 (PMC8625946; doi:10.3390/ma14226891)
Supplement: Supplementary file 1 [file materials-14-06891-s001.zip › materials-1431657-SI.pdf]

Supplementary information for

Synthesis and Characterization of Activated Carbon Co-Mixed Electrospun Titanium Oxide Nanofibers as Flow Electrode in Capacitive Deionization

Gbenro Folaranmi, Myriam Tauk, Mikhael Bechelany, Philippe Sistat, Marc Cretin* and Francois Zaviska*

Institut Européen des membranes, IEM, UMR-5635, Université de Montpellier, ENSCM, CNRS, Place Eugène Bataillon, 34095 Montpellier cedex 5, France.

Corresponding author: marc.cretin@umontpellier.fr and francois.zaviska@umontpellier.fr

1.0 EDX Mapping

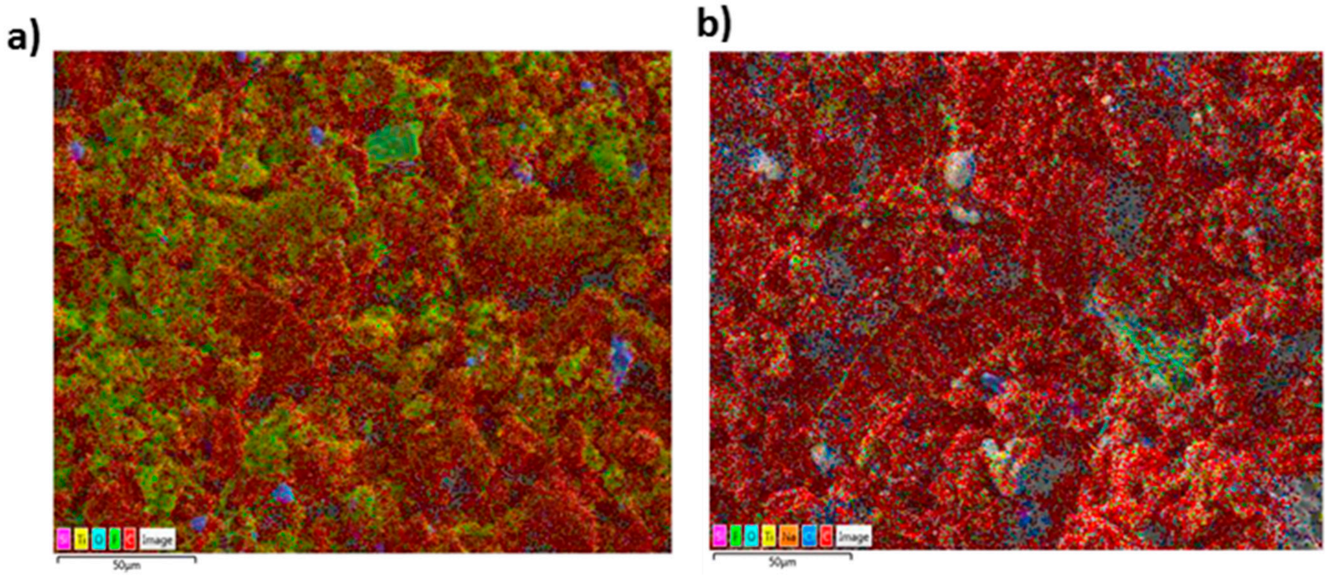

Figure S1. EDX mapping of (**a**) $ACTiO_2NFs$-5.0 and (**b**) $ACTiO_2NFs$-1.0.
